# Non-infectious immune complexes downregulate the production of interferons and tumor necrosis factor-α in primary porcine alveolar macrophages *in vitro*

**DOI:** 10.3389/fvets.2024.1420466

**Published:** 2024-06-19

**Authors:** Liujun Zhang, Xing Feng, Weizhen Chen, Bo Wang, Shaojun He, Hongjie Fan, Deyi Liu

**Affiliations:** College of Animal Science, Anhui Science and Technology University, Chuzhou, China

**Keywords:** PRRSV, NICS, IFNs, TNF-α, PAMS

## Abstract

Porcine reproductive and respiratory syndrome (PRRS) caused by the PRRS virus (PRRSV) has been harming the pig industry worldwide for nearly 40 years. Although scientific researchers have made substantial efforts to explore PRRSV pathogenesis, the immune factors influencing PRRSV infection still need to be better understood. Infectious virus-antibody immune complexes (ICs) formed by PRRSV and sub-or non-neutralizing antibodies specific for PRRSV may significantly promote the development of PRRS by enhancing PRRSV replication through antibody-dependent enhancement. However, nothing is known about whether PRRSV infection is affected by non-infectious ICs (NICs) formed by non-pathogenic/infectious antigens and corresponding specific antibodies. Here, we found that PRRSV significantly induced the transcripts and proteins of interferon-α (IFN-α), IFN-β, IFN-γ, IFN-λ1, and tumor necrosis factor-α (TNF-α) *in vitro* primary porcine alveolar macrophages (PAMs) in the early stage of infection. Our results showed that NICs formed by rabbit-negative IgG (RNI) and pig anti-RNI specific IgG significantly reduced the transcripts and proteins of IFN-α, IFN-β, IFN-γ, IFN-λ1, and TNF-α *in vitro* PAMs and significantly elevated the transcripts and proteins of interleukine-10 (IL-10) and transforming growth factor-β1 (TGF-β1) *in vitro* PAMs. NICs-mediated PRRSV infection showed that NICs not only significantly decreased the induction of IFN-α, IFN-β, IFN-γ, IFN-λ1, and TNF-α by PRRSV but also significantly increased the induction of IL-10 and TGF-β1 by PRRSV and considerably enhanced PRRSV replication *in vitro* PAMs. Our data suggested that NICs could downregulate the production of antiviral cytokines (IFN-α/β/γ/λ1 and TNF-α) during PRRSV infection *in vitro* and facilitated PRRSV proliferation in its host cells by inhibiting innate antiviral immune response. This study elucidated one novel immune response to PRRSV infection, which would enhance our understanding of the pathogenesis of PRRSV.

## Introduction

PRRSV is a single-stranded, positive-strand RNA-enveloped virus assigned to the genus *Arterivirus*, and it has two well-known species, including type 1 (European-like or PRRSV-1) represented by Lelystad and type 2 (North American-like or PRRSV-2) described by VR-2332 (1). Even though PRRSV was identified three decades ago, due to its long persistence of infection and complex pathogenic features, PRRS is at the top of global veterinary concerns. PRRSV causes acute respiratory problems (dyspnea, asthma, etc.) in young piglets and severe reproductive disturbances (abortion, stillbirth, etc.) in late pregnant sows, which results in considerable economic losses to global swine production (2, 3). The virus shows an extreme propensity for swine immune cells of monocytes/macrophages, notably porcine alveolar macrophages (PAMs). Still, it can also be cultivated using the African green monkey kidney MARC-145 cell line *in vitro* (4, 5). The innate immune system can defend against the invasion of most pathogenic microorganisms. The innate antiviral cytokines, such as interferons (IFNs) and TNF-α produced by immune macrophages in response to microbial pathogen stimulation, are critical components of the natural immune system that initiate antiviral response (6). Three types of IFNs (type I: IFN-α/β, type II: IFN-γ, and type III: IFN-λs) have been found in the body’s immune response (7). The outcome of virus infection could be affected by the induction of innate immune cytokines, including IFNs, TNF-α, IL-10, and TGF-β1. A growing number of reports demonstrate that PRRSV infection influences innate immunity by altering cytokine secretion of the host cells (8, 9). However, there are conflicting findings on the ability of PRRSV to regulate cytokine responses, and different PRRSV may differ in the capability to induce cytokine expression (10, 11). Multiple factors may be responsible for these controversial results, such as host cell types, detection methods, virus species, etc.

Macrophages can express the receptors for different immunoglobulin (Ig) molecules. The IgG Fc fragment receptors (Fc gamma receptors, FcγRs) are the most typical leukocyte phagocytic receptors (12). Antibodies (IgGs)/cognate foreign or self-antigens binding forms the immune complexes (ICs) that have long been known to modulate the host immune responses through their abilities to cluster FcγRs expressed on the surface of immune cells (13–15). The ICs-FcγRs interaction leads to antigen presentation, the release of chemokines and cytokines, phagocytosis, antibody-dependent cellular cytotoxicity, neutrophil activation and degranulation, B or T cell selection, maturation and activation (16–18). Besides regulating host immunity, the ICs have also been proven to suppress autophagy in glomerular endothelial cells, be tightly associated with many human and animal diseases, and be used in immunotherapies as an alternative treatment for several forms of tumor and in preventive/therapeutic vaccines preventing viral outbreaks in the host populations (19–21). Specific antibodies induced by the virus are usually antiviral and can prevent further viral infection in a number-dependent manner. Unfortunately, although some viruses activate a rapid humoral immune response of the host, these antibodies may enhance viral replication in target cells via antibody-dependent enhancement (ADE) of infection (22). Among the different types of viruses affected by ADE, the most notable is dengue virus (DENV), Ross River virus (RRV), Ebola virus, feline infectious peritonitis virus, Zika virus, human immunodeficiency virus type 1, and PRRSV (owing to its veterinary importance) (22, 23). Principally, FcγRs-mediated endocytosis of infectious ICs formed by viruses and preexisting sub-or non-neutralizing antibodies against viruses is responsible for the ADE (24). ADE infection promotes the entry process of infectious virus-antibody ICs by FcγRs-mediated endocytosis and modifies innate intracellular antiviral mechanisms in FcγRs-bearing cells. For instance, ADE of DENV infection down-regulates the synthesis of IFN-β/γ and TNF-α (25–27). RRV-ADE infection results in suppression of IFN-β and TNF-α (28). The decrease of IFN-α/β/γ/λs and TNF-α levels is also observed in ADE of PRRSV infection (29–31). The NICs formed in the physiological progress of antibody response to various non-pathogenic/infectious antigens widely exist in living organisms (32, 33). In addition, the combination of utterly neutralizing antibodies induced by viruses and corresponding viruses in viral infection also forms NICs. Nevertheless, whether the NICs influence the antiviral response to PRRSV infection is unknown.

This study explored the effect of artificial analog NICs formed by rabbit-negative IgG (RNI) and pig anti-RNI IgG antibodies on the innate antiviral response to PRRSV infection *in vitro* PAMs. The results might provide evidence to fill knowledge on NICs regulating virus-induced immune response, which would deepen understanding of the immune pathogenesis of PRRSV.

## Materials and methods

### Cells and virus

As described previously (34), PAMs used in this experiment were separated from nine PRRSV-negative 21 to 42-day-old pigs. The cells were maintained in Roswell Park Memorial Institute medium (HyClone, United States) supplemented with 10% fetal bovine serum (FBS) (HyClone, United States) plus streptomycin–penicillin (HyClone, United States). PRRSV-2 virus strain HeN-3 (Accession number: FJ237420) used in all experiments was propagated in MARC-145 cells cultured in Dulbecco’s modified Eagle’s medium (HyClone, United States) containing 10% FBS and streptomycin–penicillin. Viral titers (50% tissue culture infectious dose, TCID_50_) were titrated in MARC-145 cells. Briefly, PRRSV suspension (10 × diluted) was inoculated onto MARC-145 cells (80% confluence) prepared in 96-well plates (Corning, United States) and cultivated for 72 h at 37°C in 5% CO_2_. The cytopathic effect was observed using a microscope. Viral TCID_50_ titers were calculated using the Reed-Muench method (35).

### Antibodies

Rabbit-negative IgG (RNI) was purified from the blood serums of specific-pathogen-free white rabbits. Pig-negative IgG (PNI) was purified from the sera of healthy PRRSV-negative piglets. Pig anti-RNI specific polyclonal antibody (pAb) (ELISA titers: 12800) was derived from healthy PRRSV-negative piglets immunized with RNI coupled with Freund’s Adjuvant (Sigma, United States). Pig anti-RNI specific IgG was depurated from pig anti-RNI specific pAb by ammonium sulfate precipitation and diethylaminoethanol chromatography. Rabbit anti-PRRSV-N protein pAb was purchased from Bioss (Beijing) Biotechnology Co., LTD. (China). Rabbit anti-β-actin pAb and goat anti-rabbit IgG (H + L)-Horseradish peroxidase (HRP) antibodies were purchased from Absin (Shanghai) Biochemical Co., LTD. (China).

### PRRSV infection assay and NICs-mediated cytokine assay in PAMs

Pig anti-RNI IgG (1.14 mg/mL)/RNI (1.14 mg/mL) non-infectious immune complexes (NICs) and PNI (1.14 mg/mL)/RNI (1.14 mg/mL) admixtures (PNI + RNI) were prepared as previously reported (36). Briefly, 1.14 mg/mL RNI was thoroughly mixed and incubated with an equal volume of pig anti-RNI specific IgG antibodies (1.14 mg/mL) or PNI (1.14 mg/mL) at 37°C for 1 h to form NICs or PNI + RNI mixtures, respectively. IFN-α, IFN-β, IFN-γ, IFN-λ1, TNF-α, IL-10, and TGF-β1 were several key immune cytokines closely associated with innate antiviral response. To investigate whether these cytokine levels were altered by PRRSV or NICs, PAM cell monolayers (5 × 10^5^ cells) were prepared in triplicates 8 h in advance in 24-well plates (Corning, United States). Then, the cells were incubated with PRRSV (200 TCID_50_), PNI + RNI, NICs, or lipopolysaccharide (LPS) (100 ng/mL) (Sigma, United States) at 37°C for 2 h. PAMs treated with LPS served as a positive control. PAMs treated with PNI + RNI served as a negative control. The untreated cells served as mock trials. Subsequently, the inoculum was replaced using 500 μL fresh growth media. The cells and their supernatants were collected after 12, 24, 36, 48, 60, or 72 h infection to detect cytokine expression by relative quantitative RT-PCR/ELISA and PRRSV production with titration/real-time RT-PCR.

### The NICs-mediated PRRSV infection in PAMs

To further analyze whether NICs affected PRRSV-induced production of IFN-α, IFN-β, IFN-γ, IFN-λ1, TNF-α, IL-10, and TGF-β1, PAM cell monolayers (5 × 10^5^ cells) were kept in triplicates 8 h in advance in 24-well plates, then pretreated with 200 μL NICs or PNI + RNI at 37°C for 2 h. After removing the culture solutions, the treated cells were incubated with 200 TCID_50_ of PRRSV at 37°C for 2 h. The cells infected with PRRSV alone acted as control groups. The inoculum was discarded, and 500 μL complete media was added. The cells and their supernatants were harvested at 12, 24, 36, 48, 60, or 72 h post-infection to quantify cytokine expression by relative quantitative RT-PCR/ELISA and PRRSV production with titration/real-time RT-PCR.

### Relative quantitative RT-PCR of immune cytokine mRNAs

Total PAM cell RNAs were obtained using TRIzol reagent (Takara, Japan), and approximately 200 ng RNAs were converted to cDNAs by reverse transcription (RT) kits (Takara, Japan). The cDNAs were used as the relative quantitative RT-PCR template. PCR reaction with primers listed in Table 1 was performed on an Applied Biosystems QuantStudio 5 Real-Time thermocycler (United States). For PCR amplifications, 2 μL cDNA templates were added to a mixture containing 10 μL TB Green II Premix (Takara, Japan), 6 μL sterilized deionized water, 1 μL forward primer (20 pmol/μL), and 1 μL reverse primer (20 pmol/μL). The cycling conditions were 95°C for 2 min, followed by 40 cycles of 95°C for 5 s and 60°C for 20 s. Cycle threshold (CT) values were obtained at the end of PCR amplifications. The transcript of the porcine β-actin (internal reference gene) was examined to normalize the input mRNA amount. As described previously (37–39), quantitative calculation of the target gene transcript began with the difference (∆CT) between the CT values of the target gene and the internal reference gene: ∆CT = CT (target gene) - CT (internal reference gene). These relative values were transformed into absolute values using the formula: Comparative mRNA expression level = 2^−∆CT^.

**Table 1:** Relative quantitative RT-PCR primers.

Gene	Primer sequence (5′-3′)
IFN-α	Forward: GGATCAGCAGCTCAGGG Reverse: GAGGGTGAGTCTGTGGAAGTA
IFN-β	Forward: CAACAAAGGAGCAGCAAT Reverse: TGGAGCATCTCGTGGATA
IFN-γ	Forward: AGCCAAATTGTCTCCTTCTA Reverse: AAGTCATTCAGTTTCCCAGA
IFN-λ1	Forward: AACTTCAGGCTTGCATCAGG Reverse: TCTTTCTTTGTGGCTTCTTGG
TNF-α	Forward: AGCCGCATCGCCGTCTCCTAC Reverse: CCTGCCCAGATTCAGCAAAGTCC
IL-10	Forward: GCATCCACTTCCCAACCA Reverse: TCGGCATTACGTCTTCCAG
TGF-β1	Forward: GAGCCAGAGGCGGACTA Reverse: GGGTGCCCTTGAATTTATC
β-actin	Forward: CGGGACATCAAGGAGAAGC Reverse: CTCGTTGCCGATGGTGATG

### Real-time RT-PCR of PRRSV RNA copies

PRRSV RNA copies in infected cells were detected and quantified using real-time RT-PCR based on the HeN-3 ORF7 gene (37). Briefly, viral RNA was isolated with TRIzol reagent and reverse-transcribed onto cDNA using commercial RT kits. Real-time RT-PCR of PRRSV RNA was performed on the Applied Biosystems QuantStudio 5 Real-Time thermocycler using the following primer pairs (Forward primer: 5’-AAACCAGTCCAGAGGCAAGG-3′; Reverse primer: 5’-GCAA-ACTAAACTCCACAGTGTAA-3′). The cycling conditions were 95°C for 10 min, followed by 40 cycles of 95°C for 5 s and 60°C for 34 s. A standard curve was generated in each detection using the plasmid standard substances. An excellent linear relation was observed when the plasmid template was diluted from 10^1^ to 10^9^ copies/μL (correlation coefficient *R^2^* = 0.999; amplified efficiency *E* = 0.959; the minimum detection limit = 20.8 copies/μL; and variation coefficient < 2.0%). PRRSV RNA copies were calculated according to the standard curve.

### ELISA assay of immune cytokine proteins

The protein concentrations of innate immune cytokines IFN-α, IFN-β, IFN-γ, IFN-λ1, TNF-α, IL-10, and TGF-β1 in cell culture supernatants were calculated using commercial ELISA kits based on each standard curve generated using a known standard. All detections were performed in parallel. IFN-α or TNF-α ELISA kits were obtained from Sigma Corporation (United States). R&D Systems (United States) provided IFN-γ or TGF-β1 ELISA kits. IFN-β or IL-10 ELISA kits were purchased from Invitrogen (United States). IFN-λ1 ELISA Kits were the products of MyBioSource Inc., (United States).

### Western blot assay

PAMs were lysed using lysis buffer and boiled in 5 × sodium dodecyl sulfate-polyacrylamide gel electrophoresis (SDS-PAGE) loading buffer at 100°C for 10 min. Proteins were resolved by 10% SDS-PAGE and transferred onto a methanol-activated polyvinylidene difluoride (PVDF) Immobilon-P membrane (Sigma, United States). After blocking with 5% nonfat dry milk buffer, the PVDF membranes were stained by primary (rabbit anti-β-actin pAb, 1:1000 dilution; rabbit anti-PRRSV-N protein pAb, 1:1000 dilution) and secondary antibodies (goat anti-rabbit IgG-HRP antibody, 1:10000 dilution). The immunolabeled proteins were visualized using an ECL luminescence reagent (Bio-Rod, United States).

### Statistical analysis

Data were expressed as mean ± standard error of the mean (SEM) from three repeated experiments. Statistical analyses were performed to determine which group differed by pairwise multiple comparisons using a two-way analysis of variance (ANOVA) followed by Bonferroni post-tests using the GraphPad Prism 5.0 software package. A value of *p* < 0.05 was considered significant.

## Results

### PRRSV induces an early innate antiviral response in PAMs

**Figure 1:**
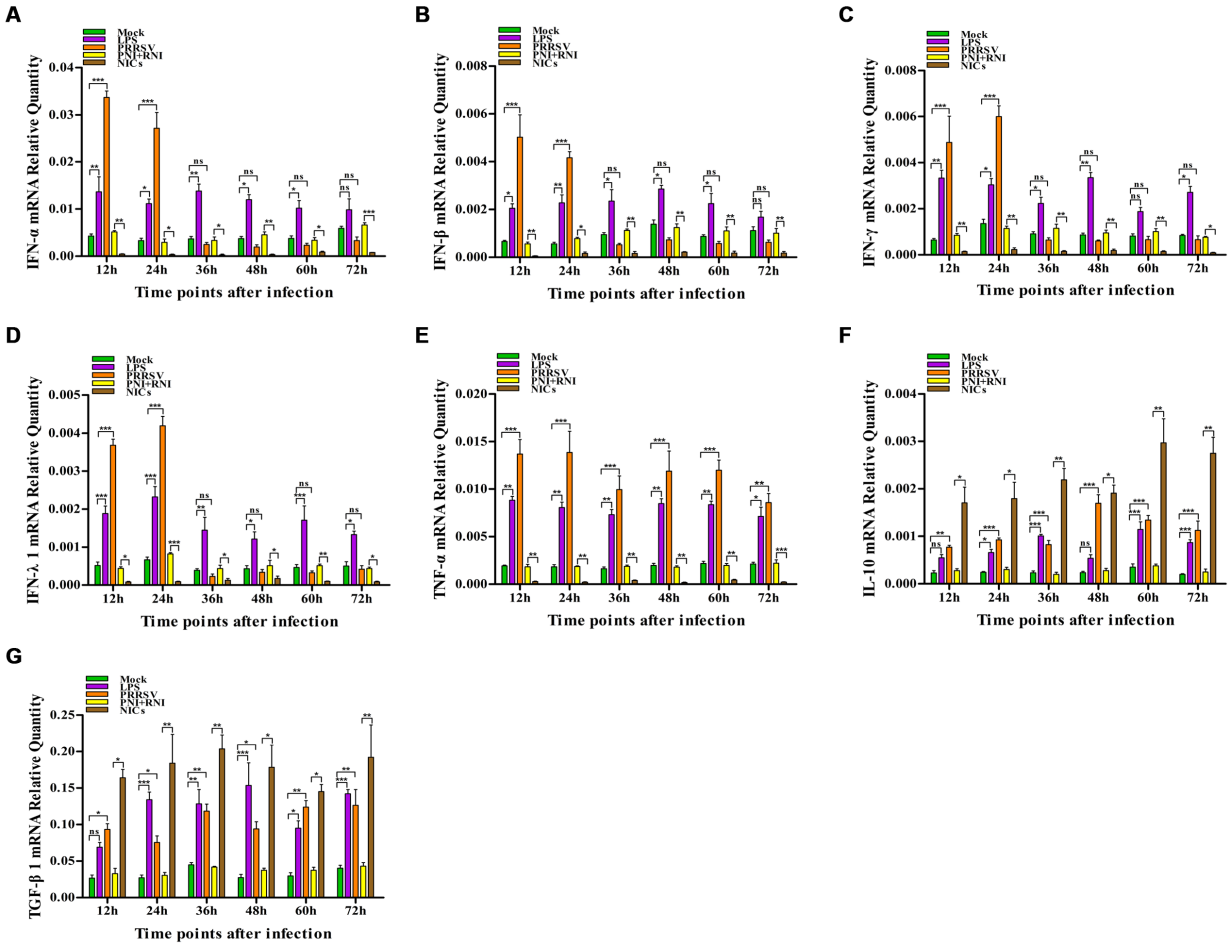
Effect of PRRSV or NICs on immune cytokine mRNAs in PAMs. PAM cell monolayers were incubated with PRRSV (200 TCID_50_), PNI + RNI, NICs, or LPS (100 ng/mL). Cells were collected for total RNA isolation at 12, 24, 36, 48, 60, or 72 h later. IFN-α **(A)**, IFN-β **(B)**, IFN-γ **(C)**, IFN-λ1 **(D)**, TNF-α **(E)**, IL-10 **(F)**, or TGF-β1 **(G)** mRNA expression was analyzed by relative quantitative RT-PCR method. β-actin was used to normalize cytokine mRNA levels. ^***^p < 0.001, ^**^p < 0.01, ^*^p < 0.05. Bars indicate the 2^−∆CT^ of mRNA copies of cytokines in cells ± SEM from three repeated experiments. LPS, lipopolysaccharide; NICs, non-infectious immune complexes; PNI, pig-negative IgG; RNI, rabbit-negative IgG; ns, no significance.

**Figure 2:**
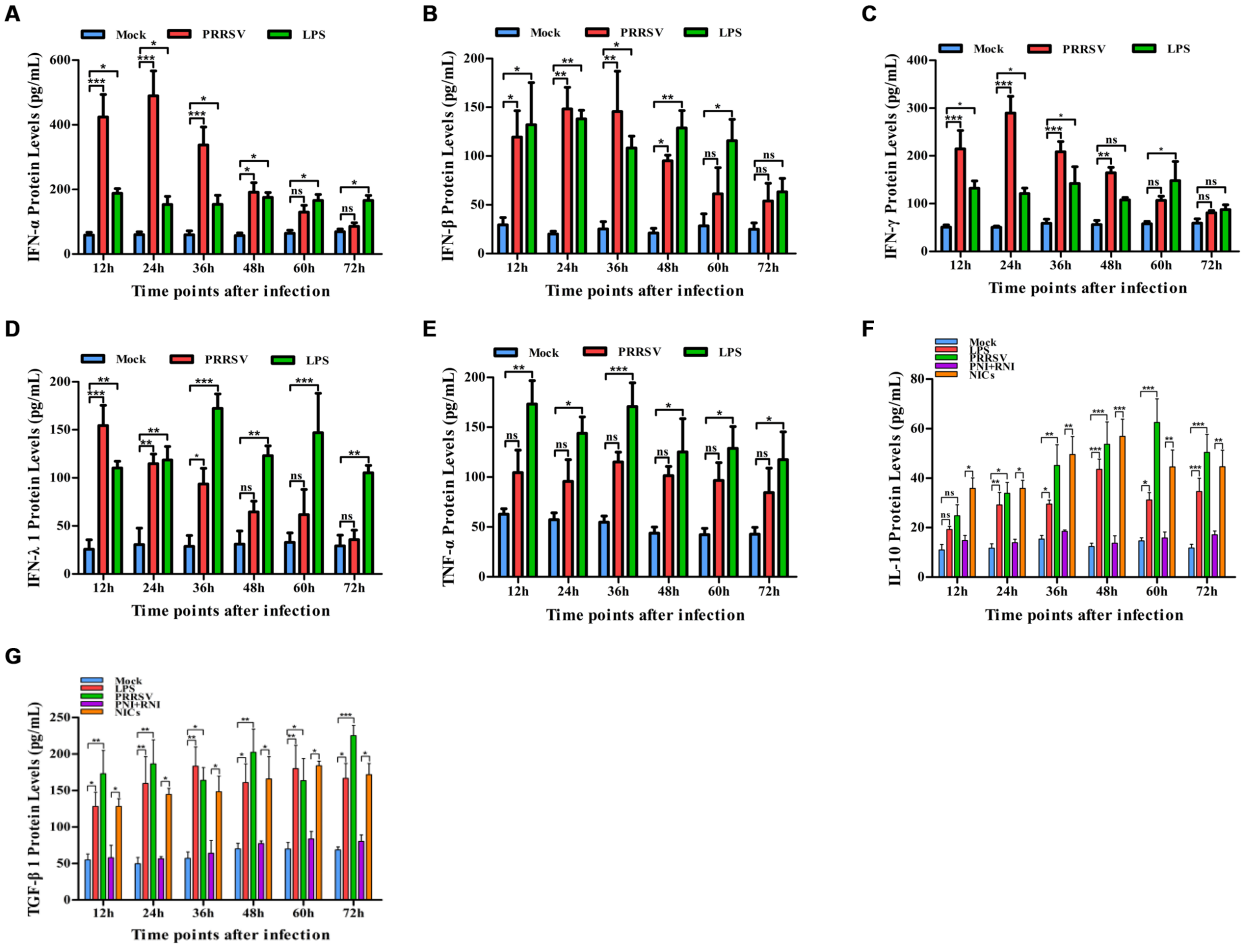
Effect of PRRSV or NICs on immune cytokine proteins in PAMs. PAM cell monolayers were incubated with PRRSV (200 TCID_50_), PNI + RNI, NICs, or LPS (100 ng/mL). Cell supernatants were collected for ELISA assay at 12, 24, 36, 48, 60, or 72 h later. IFN-α **(A)**, IFN-β **(B)**, IFN-γ **(C)**, IFN-λ1 **(D)**, TNF-α **(E)**, IL-10 **(F)**, or TGF-β1 **(G)** protein production was quantified using commercial ELISA kits. ^***^p < 0.001, ^**^p < 0.01, ^*^p < 0.05. Bars indicate the mean of protein concentrations of cytokines in cell supernatants ± SEM from three repeated experiments. LPS, lipopolysaccharide; NICs, non-infectious immune complexes; PNI, pig-negative IgG; RNI, rabbit-negative IgG; ns: no significance.

**Figure 3:**
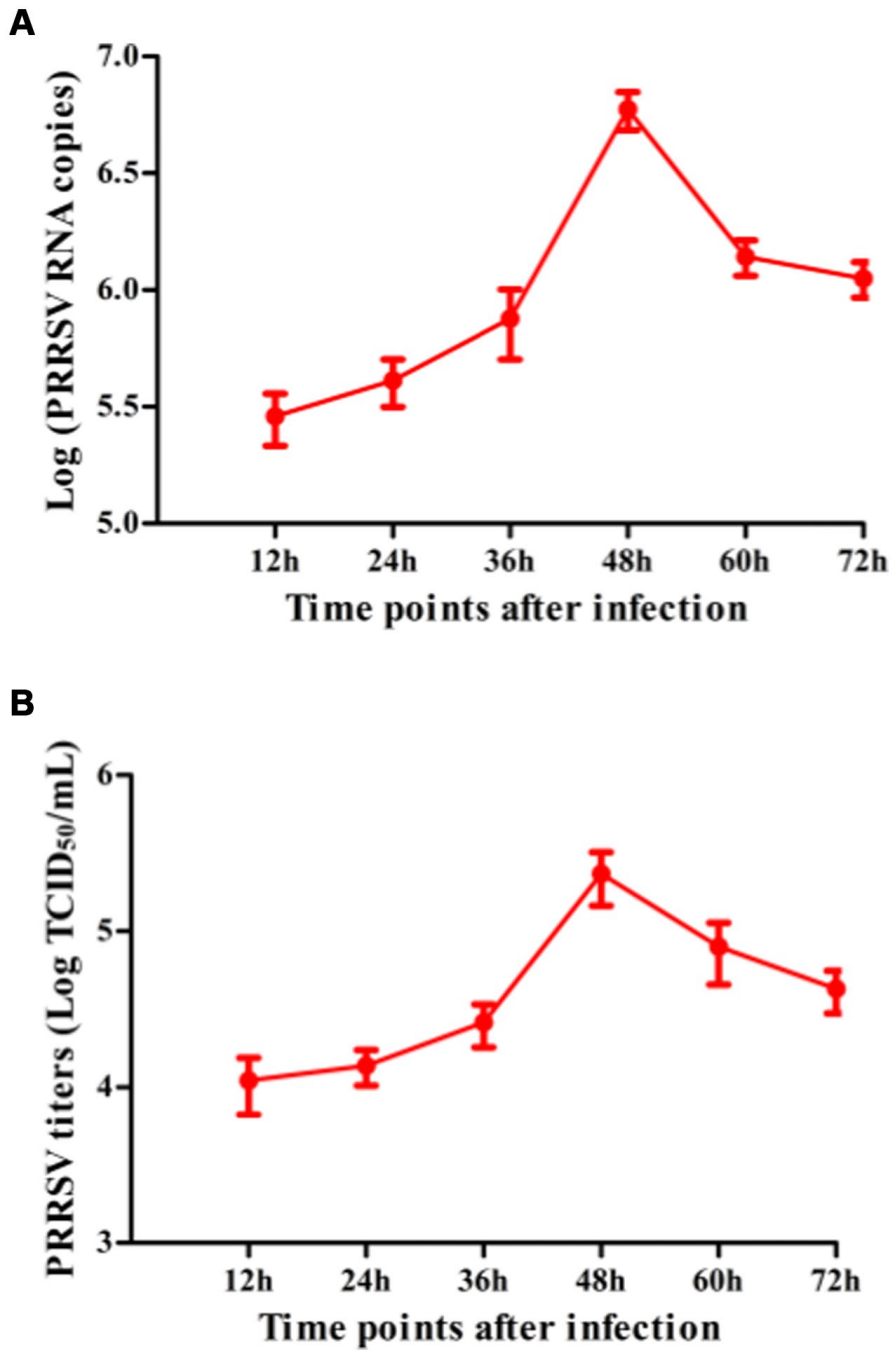
The kinetics of PRRSV proliferation in PAMs. PAM cell monolayers were infected with 200 TCID_50_ of PRRSV. Cell supernatants were harvested at 12, 24, 36, 48, 60, or 72 h post-infection. PRRSV RNA copies **(A)** and its TCID_50_/mL titers **(B)** in cell supernatants were measured by real-time RT-PCR and TCID_50_ assay. Data represents mean ± SEM of three repeated experiments.

To explore whether PRRSV induced the production of immune cytokines IFN-α, IFN-β, IFN-γ, IFN-λ1, TNF-α, IL-10, and TGF-β1, PAM cell monolayers were infected with PRRSV for 12, 24, 36, 48, 60, or 72 h. Cytokine mRNAs in infected cells and cytokine proteins in culture supernatants were measured, respectively. As illustrated in Figures 1A–D, significant up-regulation of IFN-α, IFN-β, IFN-γ, and IFN-λ1 mRNAs was detected in virus-infected cells at 12, 24 h later, and weak down-regulation of IFN-α, IFN-β, IFN-γ, and IFN-λ1 mRNAs was detected in virus-infected cells at 36–72 h later, compared to uninfected cells. Simultaneously, IFN-α, IFN-β, IFN-γ, and IFN-λ1 proteins from virus-infected cell supernatants were significantly more than those from uninfected cell supernatants at 12–48 h later and slightly more than those from uninfected cell supernatants at 60, 72 h later (Figures 2A–D). Although significant up-regulation of TNF-α mRNA was detected in virus-infected cells at 12–72 h later compared to uninfected cells, TNF-α protein from virus-infected cell supernatants was only weakly more than that from uninfected cell supernatants at 12–72 h later (Figures 1E, 2E). In addition, significant up-regulation of the mRNAs and proteins of IL-10 and TGF-β1 were detected in virus-infected cells at 12–72 h later, compared to uninfected cells (Figures 1F,G, 2F,G). PRRSV kinetics showed the highest levels of viral RNAs and TCID50 titers after 48 h infection (Figure 3). These data suggested that PRRSV induced an early innate antiviral response.

### NICs suppress innate antiviral response in PAMs

To confirm whether non-infectious immune complexes (NICs) regulated the expression of immune cytokines IFN-α, IFN-β, IFN-γ, IFN-λ1, TNF-α, IL-10, and TGF-β1, PAM cell monolayers were treated with NICs for 12, 24, 36, 48, 60, or 72 h. After collecting treated cells and culture supernatants, further cytokine mRNA quantitative analysis and cytokine protein ELISA assay were performed. As exhibited in Figures 1A–E, a significant reduction of IFN-α, IFN-β, IFN-γ, IFN-λ1, and TNF-α mRNAs was observed in NICs-treated cells after 12–72 h treatment, compared to PNI + RNI-treated cells. Concurrently, examination of NICs-treated cell supernatants showed no detectable levels for IFN-α, IFN-β, IFN-γ, IFN-λ1, or TNF-α protein. However, significant enhancement of IL-10 and TGF-β1 mRNAs in NICs-treated cells and their proteins from NICs-treated cell supernatants were observed at 12–72 h post-treatment, compared to PNI + RNI-treated cells (Figures 1F,G, 2F,G). These data suggested that NICs suppress the innate antiviral response.

### NICs repress PRRSV-induced innate antiviral response in PAMs

**Figure 4:**
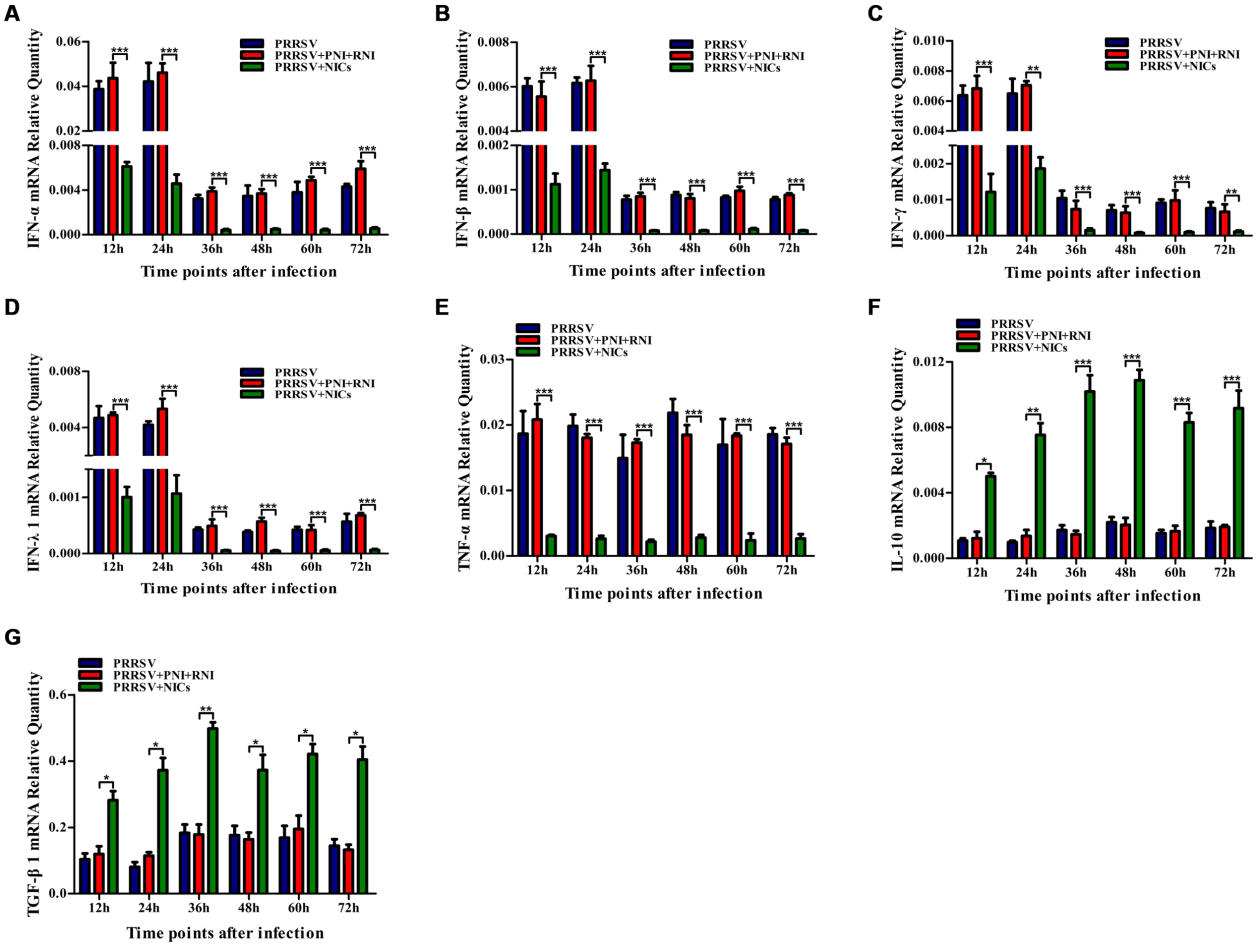
Effect of NICs on immune cytokine mRNAs in PAMs infected with PRRSV. PAM cell monolayers were pretreated with PNI + RNI or NICs for 2 h and then infected with 200 TCID_50_ of PRRSV. Cells were collected for total RNA isolation at 12, 24, 36, 48, 60, or 72 h post-infection. IFN-α **(A)**, IFN-β **(B)**, IFN-γ **(C)**, IFN-λ1 **(D)**, TNF-α **(E)**, IL-10 **(F)**, or TGF-β1 **(G)** mRNA expression was analyzed by relative quantitative RT-PCR method. β-actin was used to normalize cytokine mRNA levels. ^***^p < 0.001, ^**^p < 0.01, ^*^p < 0.05. Bars indicate the 2^−∆CT^ of mRNA copies of cytokines in cells ± SEM from three repeated experiments. NICs, non-infectious immune complexes; PNI, pig-negative IgG; RNI, rabbit-negative IgG.

**Figure 5:**
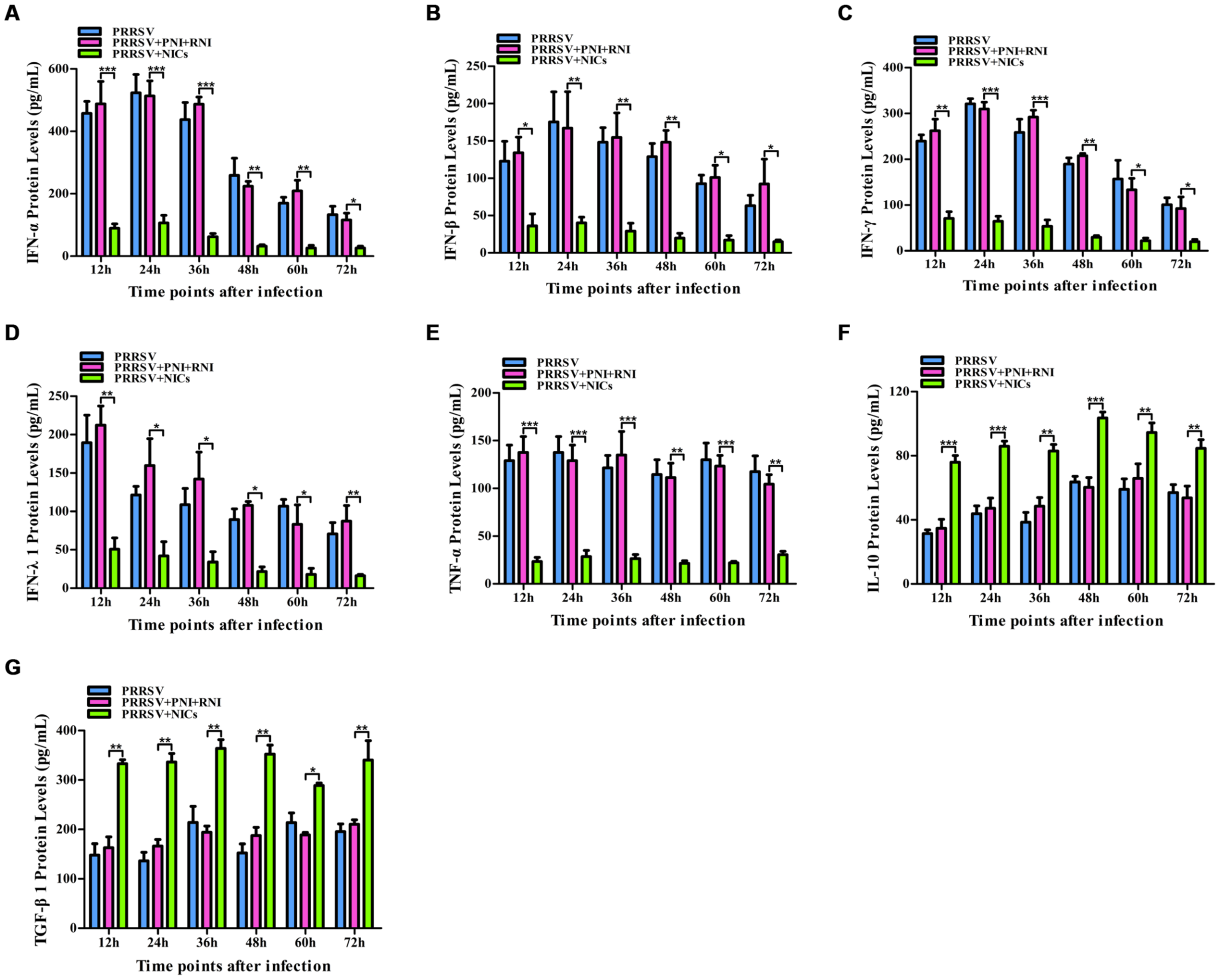
Effect of NICs on immune cytokine proteins in PAMs infected with PRRSV. PAM cell monolayers were pretreated with PNI + RNI or NICs for 2 h and then infected with 200 TCID_50_ of PRRSV. Cell supernatants were collected for ELISA assay at 12, 24, 36, 48, 60, or 72 h post-infection. IFN-α **(A)**, IFN-β **(B)**, IFN-γ **(C)**, IFN-λ1 **(D)**, TNF-α **(E)**, IL-10 **(F)**, or TGF-β1 **(G)** protein production was quantified using commercial ELISA kits. ^***^p < 0.001, ^**^p < 0.01, ^*^p < 0.05. Bars indicate the mean of protein concentrations of cytokines in cell supernatants ± SEM from three repeated experiments. NICs, non-infectious immune complexes; PNI, pig-negative IgG; RNI, rabbit-negative IgG.

To determine whether NICs influenced the secretion of IFN-α, IFN-β, IFN-γ, IFN-λ1, TNF-α, IL-10, and TGF-β1 induced by PRRSV, PAM cell monolayers were pretreated with NICs for 2 h before infecting by PRRSV for 12, 24, 36, 48, 60, or 72 h. The cells and supernatants were harvested for mRNA relative quantitative and protein ELISA analysis of immune cytokines. As shown in Figures 4, 5, the mRNAs of IFN-α, IFN-β, IFN-γ, IFN-λ1, and TNF-α in PRRSV-infected cells pretreated with NICs, and their proteins in cell supernatants were significantly decreased at 12–72 h post-infection, whereas the mRNAs of IL-10 and TGF-β1 in PRRSV-infected cells pretreated with NICs and their proteins in cell supernatants were significantly increased at 12–72 h post-infection compared with PRRSV-infected cells pretreated with PNI + RNI. These data suggested that NICs repressed PRRSV-induced innate antiviral response.

### NICs enhance PRRSV replication in PAMs

**Figure 6:**
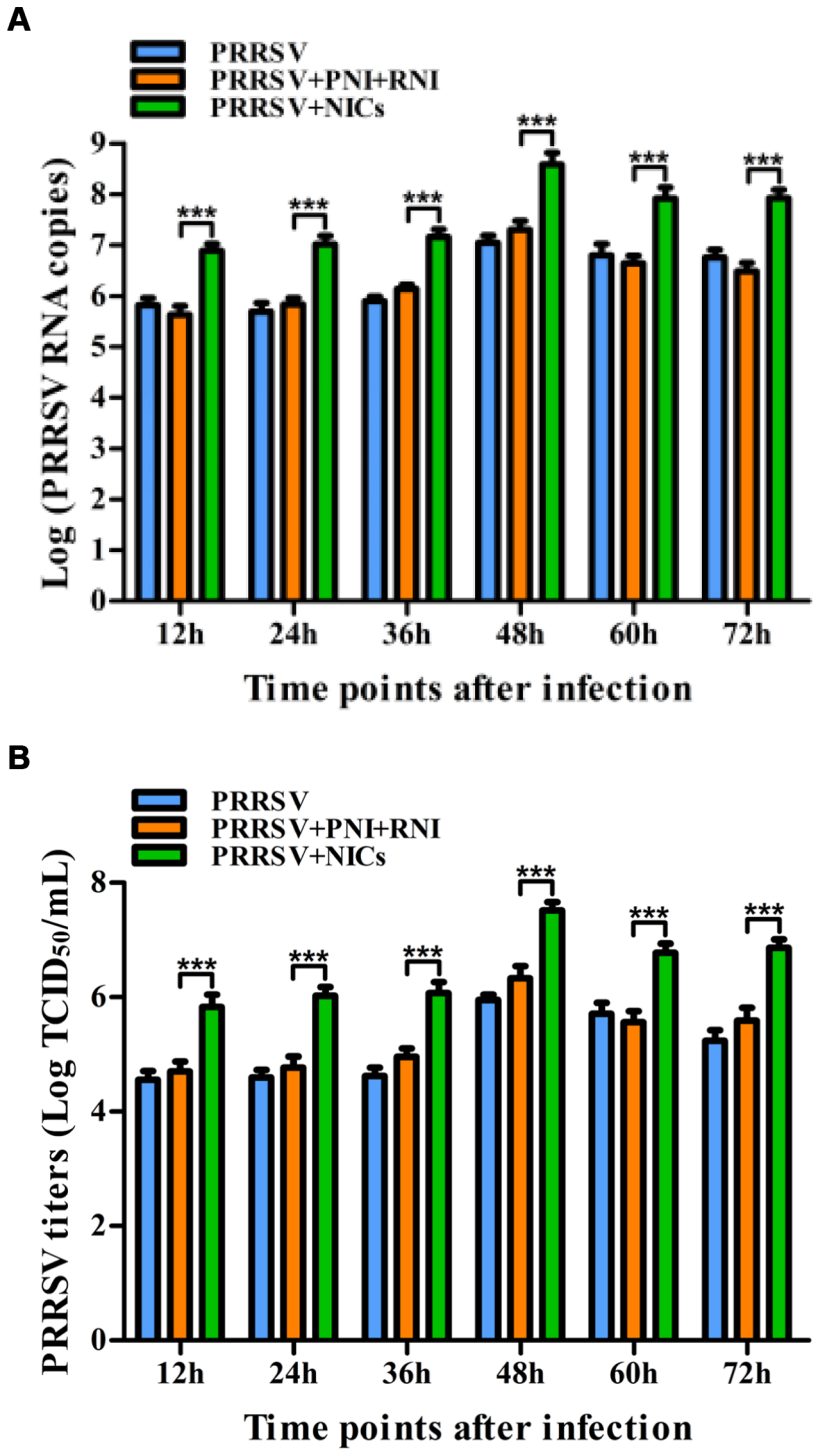
Effect of NICs on PRRSV propagation in PAMs. PAM cell monolayers were pretreated with PNI + RNI or NICs for 2 h and then infected with 200 TCID_50_ of PRRSV. Cell supernatants were harvested at 12, 24, 36, 48, 60, or 72 h post-infection. PRRSV RNA copies **(A)** and its TCID_50_/mL titers **(B)** in cell supernatants were measured by real-time RT-PCR and TCID_50_ assay. ^***^p < 0.001. Bars indicate RNA copies or TCID_50_/mL titers of PRRSV. Error bars indicate the mean ± SEM of three repeated experiments. NICs, non-infectious immune complexes; PNI, pig-negative IgG; RNI, rabbit-negative IgG.

**Figure 7:**
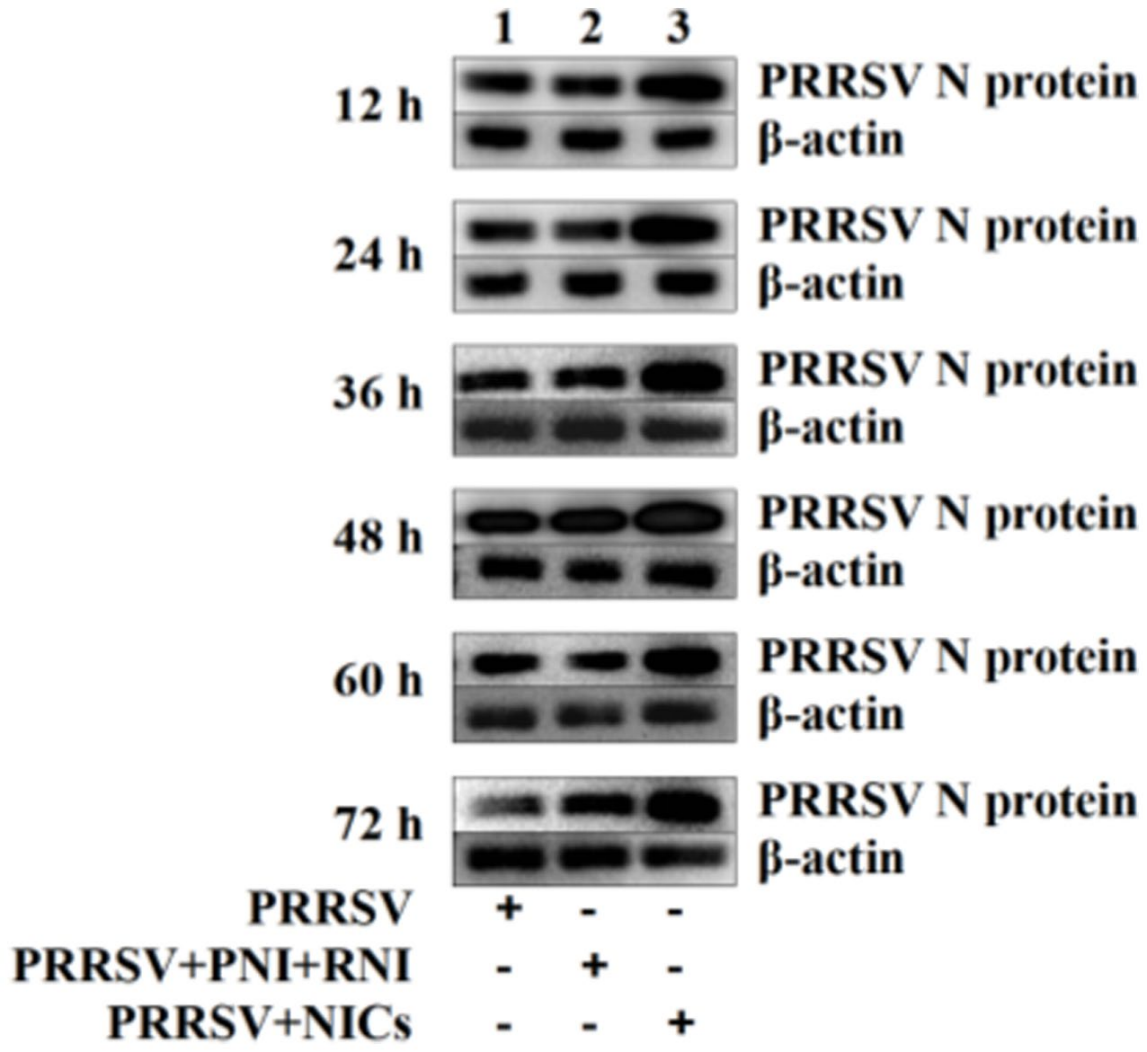
Immunoblot analysis of PRRSV N protein in PAMs. PAM cell monolayers were pretreated with PNI + RNI or NICs for 2 h and then infected with 200 TCID_50_ of PRRSV. Cells were collected for protein extraction at 12, 24, 36, 48, 60, or 72 h post-infection. Whole-cell proteins were prepared for western blotting with primary (rabbit anti-PRRSV-N protein pAb, 1:1000 dilution) and secondary antibodies (goat anti-rabbit IgG-HRP antibody, 1:10000 dilution). β-actin served as a loading control. NICs, non-infectious immune complexes; PNI, pig-negative IgG; RNI, rabbit-negative IgG; pAb, polyclonal antibody.

Since we demonstrated that NICs repressed PRRSV-induced innate antiviral response, we next test whether NICs influenced PRRSV replication. PAM cell monolayers were pretreated with NICs for 2 h before being infected by PRRSV for 12, 24, 36, 48, 60, or 72 h. The infected cell supernatants were collected to detect viral RNAs and TCID_50_ titers. Whole-cell lysates were extracted for immunoblot analysis of PRRSV N protein in PAMs by western blotting. The results seen in Figure 6 showed that the RNAs and TCID_50_ of PRRSV in supernatants of PRRSV-infected cells pretreated with NICs were significantly higher than those in supernatants of PRRSV-infected cells pretreated with PNI + RNI after 12–72 h infection. The increases in RNA copies and TCID_50_/mL titers of PRRSV by NICs ranged from 10.42–27.63 folds and 12.94–18.80 folds, respectively. Similar results were also observed in Figure 7 (lanes 2 and 3). These data suggested that NICs facilitate PRRSV replication.

## Discussion

Immune monocyte/macrophage lineages play an essential role in the innate immune system by producing many cytokines. Classically activated macrophages are pro-inflammatory by releasing pro-inflammatory cytokines such as TNF-α and IL-1β/6/12/23. Alternatively activated macrophages are anti-inflammatory and immunoregulatory by secreting anti-inflammatory cytokines such as IL-10 and TGF-β (40). The cytokine profile of target cells in response to PRRSV infection has been extensively studied. It seems generally accepted that PRRSV replication in host cells down-regulates the synthesis of innate antiviral cytokines (41, 42). Direct PRRSV replication may result in immunosuppression of the host, contributing to secondary infection or triggering other diseases whose clinical symptoms are directly related to changes in the immune system (43, 44). The inhibition of innate immunity has been considered an essential factor contributing to the PRRSV regulation of host immune responses. In this study, we explored whether PRRSV induced an antiviral response by monitoring the presence of several vital antiviral cytokines in host cells by mRNA transcription analysis and protein ELISA assay of cytokines. We found that the abundant transcripts and proteins of IFN-α, IFN-β, IFN-γ, and IFN-λ1 were detected in PRRSV-infected PAMs in early infection, and weak inhibition of IFN-α, IFN-β, IFN-γ, and IFN-λ1 was observed in PRRSV-infected PAMs in late infection, consistent with previous studies (31, 45–47). Meanwhile, a low level of TNF-α production was detected in PRRSV-infected PAMs at 12–72 h post-infection, suggesting that PRRSV was a weak inducer of TNF-α. These results indicated that PRRSV could induce the innate antiviral immune response in infected host cells by activating IFNs or TNF-α secretion. IL-10 and TGF-β1 are two important immunomodulatory cytokines that may be associated with viral infection and host immunosuppression (48, 49). Singleton et al. observed that monocyte-derived macrophages become more susceptible to PRRSV infection after pre-treament with IL-10 (50). IL-10 and TGF-β1 level up-regulation has been observed during PRRSV infection (51–53). But, there also are studies showing that PRRSV infection down-regulates or has no effect on IL-10 and TGF-β1 levels (54–56). We found that the mRNAs and proteins of IL-10 and TGF-β1 were upregulated in PRRSV-infected PAMs at any time post-infection. This evidence suggested that different PRRSV isolates may induce disparate expression patterns of IL-10 and TGF-β1.

Antibody/antigen binding generates immune complexes (ICs) with various modulatory functions. ICs play essential roles in enhanced immune activation against antigens, facilitated antigen uptake, antigen targeting/retention, antigen-presenting cell activation, and balancing stimulatory signals (21, 57). The relationship between ICs and innate antiviral response has also been described. Several early studies have shown that ICs suppress IFN-γ-induced tumoricidal activity and primary histocompatibility complex class II expression by inhibiting the transducers and activators of the Janus kinase/signal pathway (58–60). Subsequently, ICs-induced secretion of IL-6 and IL-10 in an antibody ratio-dependent manner was confirmed in human monocytes (61). Data from a recent report shows that ICs suppress IFN-γ signaling by activating the FcγRI signaling pathway (62). Furthermore, in ADE infection of viruses, including PRRSV, the cross-linking of FcγRs and infectious virus-antibody ICs formed by viruses and cognate virus-specific sub-or non-neutralizing antibodies also inhibits IFNs and TNF-α responses (29, 63). In contrast, several reports have demonstrated that ICs sometimes activate IFNs and TNF-α production (64–66). These differences may be related to the types of ICs or experimental models. However, up to date, the role of NICs formed by antibodies and all kinds of non-pathogenic/infectious antigens in innate antiviral response to virus infection has not been well understood. Previous reports showed that macrophages pre-treated with type I or II IFNs were less susceptible to PRRSV infection (50, 67). In the current study, we found that the transcripts and proteins of IFN-α, IFN-β, IFN-γ, IFN-λ1, and TNF-α in PAMs were reduced by NICs treatment, and the transcripts and proteins of IL-10 and TGF-β1 in PAMs were enhanced by NICs treatment. We showed that NICs suppressed innate antiviral response. More importantly, our data showed that NICs treatment of PAMs not only down-regulated PRRSV-induced secretion of IFN-α, IFN-β, IFN-γ, IFN-λ1, and TNF-α but also upregulated PRRSV-induced secretion of IL-10 and TGF-β1 and enhanced PRRSV replication in PAMs. These results suggested that NICs repressed innate antiviral response to PRRSV infection *in vitro.* Our studies supplied a better understanding of the interaction between NICs and host cells, primarily focusing on the effect of NICs on PRRSV replication. Further exploration of the mechanisms of NICs inhibiting innate antiviral immunity will significantly improve our knowledge of PRRSV-persistent pathogenesis and provide insights into developing novel anti-PRRSV strategies.

## Conclusion

PRRSV induced an early antiviral response by activating the secretion of IFN-α, IFN-β, IFN-γ, IFN-λ1, and TNF-α *in vitro* PAMs. NICs inhibited PRRSV-induced antiviral response by reducing PRRSV-induced synthesis of IFN-α, IFN-β, IFN-γ, IFN-λ1, and TNF-α *in vitro* PAMs and increasing PRRSV-induced production of IL-10 and TGF-β1 *in vitro* PAMs, therefore promoting PRRSV replication. Our findings provided a new insight into PRRSV infection that might be enhanced by the formation of non-pathogenic/infectious antigens-antibodies ICs, which facilitated an understanding of the immune pathogenesis of PRRSV-persistent infection.

## Data availability statement

The original contributions presented in the study are included in the article/supplementary material, further inquiries can be directed to the corresponding authors.

## Ethics statement

The animal studies were approved by Institutional Animal Care and Use Committee of Anhui Science and Technology University, China (approval no. 2022002). The studies were conducted in accordance with the local legislation and institutional requirements. Written informed consent was obtained from the owners for the participation of their animals in this study.

## Author contributions

LZ: Writing – review & editing, Writing – original draft, Investigation, Funding acquisition. XF: Writing – review & editing, Investigation. WC: Writing – review & editing, Resources. BW: Writing – review & editing, Resources. SH: Writing – review & editing, Resources. HF: Writing – review & editing, Supervision, Funding acquisition. DL: Writing – review & editing, Supervision.
